# Lower baseline scores best predict achievement of the minimal clinically important difference after hip arthroscopy: A machine learning analysis from the Femoroacetabular Impingement RandomiSed Controlled Trial and embedded prospective cohort

**DOI:** 10.1002/ksa.70053

**Published:** 2025-09-09

**Authors:** Prushoth Vivekanantha, Jeffrey Kay, Nicole Simunovic, Olufemi R. Ayeni, the FIRST Investigators

**Affiliations:** ^1^ Department of Surgery, Division of Orthopaedic Surgery McMaster University Hamilton Ontario Canada

**Keywords:** artificial intelligence, FAI, hip arthroscopy, machine learning, predictive modelling

## Abstract

**Purpose:**

This analysis evaluated whether logistic regression and machine learning models could predict achievement of the minimal clinically important difference (MCID) for the International Hip Outcome Tool (iHOT‐12) and Hip Outcome Score (HOS) at 6 and 12 months following hip arthroscopy.

**Methods:**

Data from the multicenter Femoroacetabular Impingement RandomiSed controlled Trial and its embedded prospective cohort were used. A total of 309 patients (mean ± SD age 34.0 ± 8.7 years, 37.7% female) were included. The MCID thresholds for iHOT‐12 and HOS were calculated using a distribution‐based method and were 9.0 and 13.0, respectively. Predictive models were trained with demographic, radiographic, and intraoperative variables using a 70:30 training‐to‐test data split. MCID achievement was defined as a change from preoperative to postoperative scores that surpassed the calculated threshold. Model discrimination was assessed using the area under the curve (AUC), and calibration was evaluated via slope, intercept, and Brier scores.

**Results:**

Achievement rates were 83.3% at 6 months and 81.1% at 12 months for iHOT‐12, and 64.3% at 6 months and 75% at 12 months for HOS. Logistic regression performed best at 12 months (AUC = 0.724) for iHOT‐12 with poor calibration (slope = 2.19). AUCs for HOS ranged between 0.672–0.715 at 6 months and 0.665–0.699 at 12 months. Best calibration was achieved by Least Absolute Shrinkage and Selection Operator (slope = 1.270, intercept = –0.177) at 6 months and by logistic regression at 12 months (slope = 1.093, intercept = –0.079). Lower baseline patient‐reported outcome measures (PROMs) were associated with MCID achievement in most models.

**Conclusion:**

The most robust predictor of MCID achievement for both PROMs were lower baseline scores, and can be used as a prognostic variable for preoperative counselling. Model performance for predicting MCID was superior for HOS relative to iHOT‐12. Machine learning models generally had comparable discrimination and calibration scores to traditional logistic regression models.

**Level of Evidence:**

Level III.

**Abbreviations**
AI: artificial intelligence
AUC: area under the (receiver operating) curve
BMI: body mass index
CI: confidence interval
EPV: events per variable
FAI: femoroacetabular impingement
FIRST: Femoroacetabular Impingement RandomiSed Trial
Hip Outcome Score: Hip Outcome Score
iHOT‐12: International Hip Outcome Tool
LASSO: Least Absolute Shrinkage and Selection Operator
LCEA: lateral centre edge angle
MCID: minimal clinically important difference
MRI: magnetic resonance imaging
NA: not applicable
OR: odds ratio
PASS: patient acceptable symptom state
RCT: randomized control trial
SD: standard deviation
SHAP: SHapley Additive exPlantations
VAS: Visual Analogue Scale
XGBoost: Extreme Gradient Boosting

## INTRODUCTION

Hip arthroscopy for femoroacetabular impingement (FAI) is an increasingly common procedure, with patient‐reported outcomes (PROMs) being a common marker of tracking surgical success [31]. Two hip‐specific PROMs include the International Hip Outcome Tool (iHOT‐12) and the Hip Outcome Score (HOS). The iHOT‐12 is a validated short‐form version of the original 33‐item iHOT, used to assess changes after treatment for young, active patients with hip disorders [9]. Furthermore, the HOS has been shown to have strong reliability and responsiveness to clinical improvement after arthroscopic hip surgery for FAI [17].

The Femoroacetabular Impingement RandomiSed controlled Trial (FIRST) was designed as a multicenter, blinded randomized controlled trial (RCT) comparing arthroscopic osteochondroplasty (with or without labral repair) and arthroscopic lavage (with or without labral repair) for patients with symptomatic FAI [5]. Reoperation rates were significantly lower in the osteochondroplasty group, and there were no differences in the main PROM assessed, the Visual Analogue Scale (VAS) [5]. However, other PROMs including the iHOT‐12 and HOS were also assessed [5]. The findings from the FIRST trial highlight the need to understand which patients achieve clinical improvement after hip arthroscopy.

The minimal clinically important difference (MCID) is a concept that is critical to interpreting PROMs, representing the smallest change in scores that patients perceive as beneficial or important [7, 10, 31]. MCIDs can be determined via anchor‐based methods (using patient questionnaires regarding satisfaction) or via distribution‐based methods (using statistical‐based methods to calculate values) [11]. Identifying who may achieve the MCID after various surgical procedures is important for setting patient expectations preoperatively. Recent literature has applied various predictive models to achieving the MCID after hip arthroscopy for FAI. This study aims to add to the current literature in determining whether baseline patient characteristics and intraoperative variables can predict the achievement of clinically significant outcomes. Specifically, this study aims to evaluate whether logistic regression and machine learning models could predict achievement of the MCID for the iHOT‐12 and HOS at 6 and 12 months following hip arthroscopy. It is hypothesized that both machine learning and logistic regression will be able to predict MCID achievement moderately.

## METHODS

This analysis adhered to the Transparent Reporting of a multiple variable prediction model for Individual Prognosis Or Diagnosis (TRIPOD) guidelines [3].

## FIRST

FIRST is an international multicenter RCT that recruited patients with symptomatic FAI between 18 and 50 years of age. There were two treatment arms: arthroscopic lavage with or without labral repair versus arthroscopic osteochondroplasty with or without labral repair of the hip. The complete protocol, statistical analysis plan, and the primary results of the FIRST trial have been published [5, 6, 26]. The protocol was registered at clinicaltrials.gov (NCT01623843) and ethics approval was granted to the Methods Centre at McMaster University as well as at each participating site.

Inclusion criteria included (1) documented period of nonoperative management including hip‐focused physical therapy for at least 6 months, (2) between the ages of 18 and 50 years with cam‐ or mixed‐type FAI diagnosed using radiography and/or magnetic resonance imaging (MRI), and (3) obtained temporary pain relief from a diagnostic intra‐articular hip injection. Exclusion criteria included (1) previous inclusion of the participant in a study involving FAI, (2) evidence of hip dysplasia (lateral centre edge angle or LCEA below 20 degrees), (3) advanced arthritis as per the Tönnis Grade 2 or 3, (4) presence of hip syndromes (concurrent non‐FAI related pathology), (5) previous trauma to the affected hip, (6) previous surgery on the ipsilateral or contralateral hip, (7) severe acetabular deformities (e.g., acetabular protrusio, coxa profunda, circumferential labral ossification), (8) immunosuppressive medication use, (9) chronic pain syndrome, (10) significant medical comorbidities (requiring assistance for daily activities of daily living [ADLs], (11) history of paediatric hip disease (Legg‐Calve‐Perthes; slipped capital femoral epiphysis), (12) ongoing litigation or compensation claims secondary to hip problems, and (13) any other reasons given, in the surgeon's judgement, to exclude the patient.

A total of 214 patients were included between October 2012 and November 2017 [5, 6, 26]. The iHOT‐12 is a PROM designed to assess hip‐specific health‐related quality of life, ranging from 0 (worst function) to 100 (best function), with greater values indicating better hip‐related quality of life [9]. The HOS ranges from 0 (worst function) to 100 (normal function), evaluating hip‐specific function in both ADL and sports [24].

### FIRST embedded prospective cohort

A concurrently enroled cohort of 110 patients between May 2015 and April 2018 in four trial sites in Canada and Finland who either did not meet the prior inclusion criteria or those who refused to participate in the trial. All patients received osteochondroplasty and were followed for the same outcomes as the FIRST trial (Supporting Information S1: Figure S1) [5, 6, 26].

### Surgical intervention and rehabilitation

Patients received either labral repair with either arthroscopic osteochondroplasty or lavage with normal saline. All patients followed a standardized postoperative protocol for protected weight‐bearing, pain management, and physical therapy. Patients were followed at the 2‐week, 6‐week, 3‐month, 6‐month and 12‐month period for all outcomes aside from adverse events, which were tracked up to 24 months. Full details are found in the previous trial protocols [5, 6, 26].

### Outcome for prediction

Achievement of the MCID for the iHOT‐12 and HOS scores at 6 and 12 months postoperative was the primary outcome of interest. A distribution‐based method was used to calculate the MCID, defined as one‐half of the standard deviation of iHOT‐12 and HOS scores. This approach is used to estimate the MCID when anchor‐based methods are not available.

### Predictive variables

Predictor variables were selected a priori based on clinical relevance, availability across both the RCT and the prospective cohort, and prior evidence in the literature demonstrating associations with postoperative outcomes after hip arthroscopy. Specifically, demographic variables included age, sex, body mass index (BMI), Tönnis grade, alpha angle, LCEA, presence of labral tears, outerbridge classification, sporting activity, traction time, if capsular closure was performed, if osteochondroplasty was performed, baseline iHOT‐12 (for postoperative iHOT‐12 prediction), and baseline HOS scores (for HOS prediction).

To minimize the risk of overfitting, established methodological guidelines of maintaining at least 10 events per predictor variable (EPV) for logistic regression models and at least 5–7 EPV for machine learning models was followed, which are generally more robust to overfitting due to embedded regularization techniques [21, 30]. Although the 10 events per predictor variable is a commonly cited benchmark, previous literature has shown acceptable model performance in regards to overfitting with as few as 5–9 EPV [30]. Given 324 total patients from the prospective cohort and the definitive FIRST trial and an 80% response rate of outcomes (approximately 259 patients), it was anticipated that there would be at least 50% patients who achieved the MCID given previous reports of achievement in the literature [15, 16, 22, 25]; therefore, 16 predictive variables were chosen.

### Predictive modelling

Four models were trained to predict achievement of the MCID in VAS and EuroQol‐5 dimensions scores at 6 and 12 months postoperatively: Logistic Regression, Least Absolute Shrinkage and Selection Operator (LASSO) Logistic Regression, Random Forest Classifier, Extreme Gradient Boosting (XGBoost). Missing data were assumed to be random and appropriate for multiple imputation by chained equations. If more than 50% of predictor variables were missing, the patient was removed from the dataset. For each outcome (6 and 12‐month achievement of the MCID), data were randomly split into training (70%) and test (30%) sets. LASSO hyperparameters were optimized using 10‐fold cross‐validation.

### Model performance metrics and interpretation

A 70:30 training:test split was utilized. The primary outcome was model discrimination was assessed using area under the receiver operating characteristic curve (AUC). Secondary outcomes included model calibration, which was assessed using the calibration slope (ideal = 1) and intercept (ideal = 0), both summarizing the agreement between predicted probabilities and observed outcomes. Another secondary outcome was model accuracy, which was also quantified with the Brier score, measuring mean squared error between predicted probabilities and actual outcomes (ideal = 0).

Feature importance scores were extracted from the Random Forest and XGBoost models. These scores quantify the relative contribution of each predictor to the model's decision‐making process but do not provide information on directionality. The SHapley Additive exPlanations (SHAP) is a method that can be used for both the magnitude and direction of individual predictor effects; however, given the small sample size, this was not done to prevent unstable or unreliable estimates.

### Statistical analysis

This machine learning and logistic regression analysis was focused on prediction rather than hypothesis testing; therefore, a formal power analysis was not required or performed. For logistic regression models, predictor coefficients and odds ratios (ORs) were reported to indicate the association between each predictor and the likelihood of achieving the MCID. Ninety‐five percent confidence intervals (95% CI) and *p*‐Values were calculated using the Wald method to assess the precision and statistical significance of each estimate. A list of definitions of machine learning terms is provided in Supporting Information S1: Table 1.

## RESULTS

Among 324 total patients encompassing the FIRST trial and the Embedded Prospective Cohort study, 309 patients had either a 6‐ or 12‐month score for either the iHOT‐12 or HOS score. The mean age of the 309 patients mean (SD) age of 34.0 (8.7) years (*n* = 302). There were 117 (37.7%) total female patients. A total of 30 patients were from the embedded prospective cohort. The mean (SD) BMI was 26.8 (4.6), and 277 (89.4%) patients had labral tears. There were 150 patients with a Tönnis grade of 1 (48.4%). The mean (SD) alpha angle was 63.7 (11.0) degrees. The majority of patients had an Outerbridge grade of 1 (110/307; 35.5%) and engaged in a moderate amount of activity (110/250; 38.7%). The mean (SD) traction time was 46.7 (21.3) min (*n* = 293). A total of 205 (70.7%) received osteochondroplasty and 207 (71.3%) received capsular closure.

The mean (SD) baseline iHOT‐12 score was 33.4 (18.8) among 238 patients, and the MCID was calculated to be 9.4. The mean (SD) iHOT‐12 score at 6 and 12 months postoperatively was 67.1 (26.4) and 69.8 (26.4) among 198 and 217 patients, respectively. At 6 and 12 months, 165 (83.3%) and (81.1%) achieved the MCID. The mean (SD) baseline HOS score was 37.2 (26.1) among 283 patients, and the MCID was calculated as 13.0. The mean (SD) HOS score at 6 and 12 months postoperatively was 63.1 (29.1) and 72.1 (28.1), respectively. At 6 and 12 months of 242 and 251 patients, 155 (64.3%) and 189 (75%) achieved the MCID, respectively (Table 1).

**Table 1:** Demographics and outcomes.

Total number of patients	309
Mean (SD) age (years) (*n* = 302)	34.0 (8.7)
Female (*n*)	117 (37.7%)
Mean (SD) BMI (kg/m^2^)	26.8 (4.6)
Tönnis grade	
Grade 0	122 (39.4%)
Grade 1	150 (48.4%)
Grade 2	34 (11.0%)
Grade 3	3 (1.0%)
Labral tear?	
No	32 (10.6%)
Yes	277 (89.4%)
Mean (SD) alpha angle (degrees)	63.7 (11.0)
Mean (SD) LCEA (degrees)	33.5 (6.1)
Outerbridge classification (*n* = 307)	
Grade 0	49 (15.9%)
Grade 1	110 (35.8%)
Grade 2	78 (25.4%)
Grade 3	42 (13.7%)
Grade 4	28 (9.1%)
Sporting activity (*n* = 250)	
None	0 (0%)
Light	80 (32%)
Moderate	110 (44%)
Vigorous	60 (24%)
Mean (SD) traction time (min)	46.7 (21.3)
Capsular closure performed? (*n* = 309)	
No	207 (71.4%)
Yes	102 (28.6%)
Osteochondroplasty performed?	
No	104 (33.7%)
Yes	205 (66.3%)
Mean (SD) baseline iHOT‐12 (*n* = 238)	33.4 (18.8)
iHOT‐12 scores at 6 months (*n* = 198)	67.1 (26.4)
iHOT‐12 scores at 12 months (*n* = 217)	69.8 (26.9)
Mean (SD) baseline HOS (*n* = 283)	37.2 (26.1)
HOS scores at 6 months (*n* = 290)	63.1 (29.1)
HOS scores at 12 months (*n* = 264)	72.1 (28.1)
MCID iHOT‐12 (½ SD of baseline scores)	9.0
MCID HOS (½ SD of baseline scores)	13.0
Achievement of MCID at 6 months for iHOT‐12	165 (83.3%)
Achievement of MCID at 12 months for iHOT‐12	176 (81.1%)
Achievement of MCID at 6 months for HOS	155 (64.3%)
Achievement of MCID at 12 months for HOS	189 (75%)

Abbreviations: BMI, body mass index; HOS, hip outcome score; iHOT‐12, international hip outcome tool; LCEA, lateral centre edge angle; MCID, minimal clinically important difference; SD, standard deviation.

### Model performance

#### iHOT‐12—Achievement of MCID

##### 6 months

**Figure 1:**
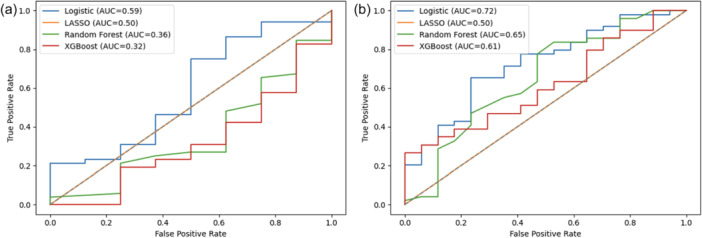
Receiver operator curves demonstrating area under the curve (AUC) for discriminatory performance of predictive models trained to predict achievement of the minimal clinically important difference (MCID) in International Hip Outcome Tool (iHOT‐12) at (a) 6 months (left) and (b) 12 months (right).

Model performance was poor for all algorithms, with logistic regression demonstrating the highest discrimination with an AUC of 0.589. However, the calibration slope and intercept were 0.077 and 0.815, respectively. LASSO failed to produce a meaningful model (AUC = 0.5, slope = 0), and Random Forest and XGBoost also had poor discriminatory performance with AUCs of 0.358 and 0.320, respectively (Table 2 and Figure 1a).

**Table 2:** Model performance.

Outcome	Model	AUC	Calibration slope	Calibration intercept	Brier score
Achievement of MCID for iHOT‐12 at 6 months	Logistic regression	0.589	0.077	0.815	0.114
	LASSO	0.5	0	0.833	0.139
	Random forest	0.358	‐0.345	1.44	0.152
	XGBoost	0.320	‐0.166	1.058	0.163
Achievement of MCID for iHOT‐12 at 12 months	Logistic regression	0.724	2.190	–1.096	0.179
	LASSO	0.5	0	0.811	0.153
	Random forest	0.651	1.775	–0.73	0.181
	XGBoost	0.612	1.075	–0.119	0.214
Achievement of MCID for HOS at 6 months	Logistic regression	0.672	0.349	0.503	0.210
	LASSO	0.715	1.270	–0.177	0.196
	Random forest	0.673	0.764	0.116	0.200
	XGBoost	0.690	0.541	0.413	0.239
Achievement of MCID for HOS at 12 months	Logistic regression	0.665	1.093	–0.079	0.178
	LASSO	0.5	0	0.75	0.188
	Random forest	0.694	0.829	0.133	0.175
	XGBoost	0.699	0.545	0.311	0.198

Abbreviations: AUC, area under the curve; HOS, hip outcome score; iHOT‐12, international hip outcome tool; LASSO, Least Absolute Shrinkage and Selection Operator; MCID, minimal clinically important difference.

##### 12 months

Model performance improved at 12 months, with logistic regression having the highest AUC of 0.724. However, the calibration slope was 2.190 and –1.096, respectively, and the Brier score was 0.179. Random Forest and XGBoost had AUCs of 0.651 and 0.612 and slopes of 1.775 and 1.075, respectively. LASSO failed to contribute a valid model (AUC = 0.5, slope = 0) (Table 2 and Figure 1b).

### HOS—Achievement of MCID

#### 6 months

**Figure 2:**
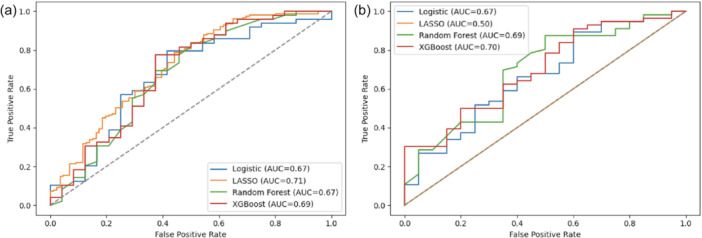
Receiver operator curves demonstrating area under the curve (AUC) for discriminatory performance of predictive models trained to predict achievement of the minimal clinically important difference (MCID) in Hip Outcome Score (HOS) at (a) 6 months (left) and (b) 12 months (right).

LASSO demonstrated the highest discriminatory performance with an AUC of 0.715, and the best calibration, with slope and intercepts of 1.270 and –0.177, respectively. All models had an AUC greater than 0.672; however, slopes for XGBoost and Random Forest were 0.541 and 0.764, with intercepts of 0.413 and 0.116, respectively. Brier scores were comparable between models (range: 0.196–0.239) (Table 2 and Figure 2a).

#### 12 months

XGBoost and Random Forest both demonstrated the highest AUCs with values of 0.699 and 0.694, respectively. However, Random Forest had better calibration with slopes, intercepts, and Brier scores of 0.829, 0.133 and 0.175, respectively. Logistic regression demonstrated similar discriminatory performance with an AUC of 0.665; however, it had the best calibration slope and intercept, with values of 1.093 and –0.079, respectively (Table 2 and Figure 2b).

### Feature importance

#### iHOT‐12—MCID predictors

##### 6 months

**Figure 3:**
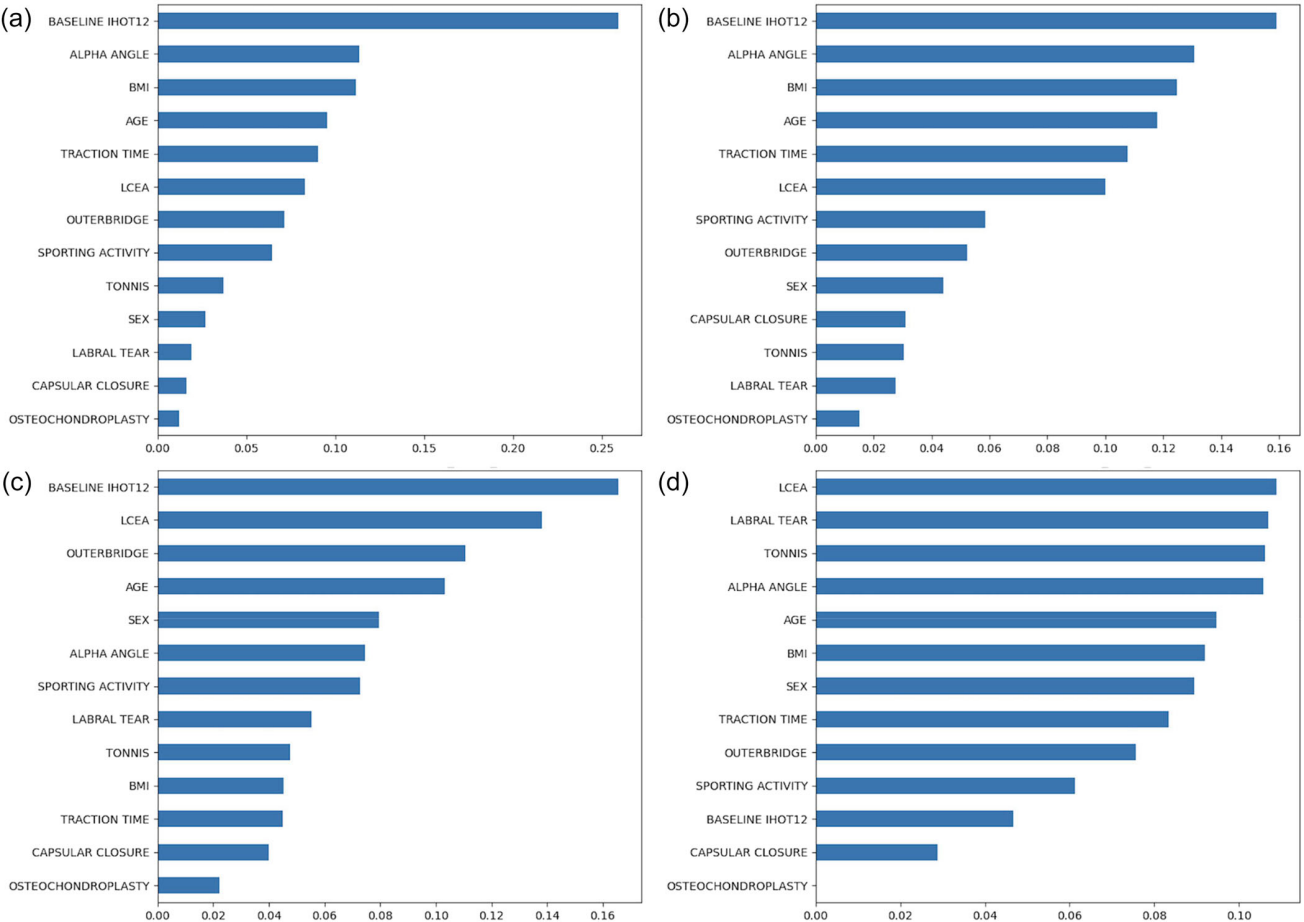
Relative feature importance for Random Forest model predicting minimal clinically important difference (MCID) of the International Hip Outcome Tool (iHOT‐12) at (a) 6 (top left) and (b) 12 (top right) months and XGBoost predicting minimal clinically important difference (MCID) of the iHOT‐12 at (c) 6 (bottom left) and (d) 12 (bottom right) months. Increasing scores refer to increasing importance.

Despite poor performance for iHOT‐12 at 6 months across all models, logistic regression performed the best, with lower baseline iHOT‐12 scores (odds ratio [OR] = 0.963, 95% confidence interval [CI]: 0.943–0.984, *p* = 0.001) and absence of osteochondroplasty (OR = 0.210, 95% CI: 0.070–0.634, *p* = 0.006) (Tables 3, 4 and Figure 3).

**Table 3:** Feature importance for top 5 features in LASSO and machine learning models.

	LASSO	Random forest	XGBoost
Achievement of MCID for iHOT‐12 at 6 months	NA^a^	Baseline iHOT‐12: 0.260 BMI: 0.126 Age: 0.113 Alpha Angle: 0.101 Traction Time: 0.092	Baseline iHOT‐12: 0.166 LCEA: 0.138 Outerbridge: 0.111 Age: 0.103 Sex: 0.080
Achievement of MCID for iHOT‐12 at 12 months	NA^a^	Baseline iHOT‐12: 0.159 Alpha angle: 0.131 BMI: 0.125 Age: 0.117 Traction Time: 0.107	LCEA: 0.109 Labral tear: 0.107 Tonnis grade: 0.106 Alpha angle: 0.106 Age: 0.095
Achievement of MCID for HOS at 6 months	Baseline HOS: ‐0.705 Capsular closure: ‐0.176 Sex: 0.118 Osteochondroplasty: ‐0.095 Traction time: 0.090	Baseline HOS: 0.179 BMI: 0.146 Age: 0.126 Alpha angle: 0.114 LCEA: 0.104	Baseline HOS: 0.140 Age: 0.088 Capsular closure: 0.084 BMI: 0.082 Outerbridge classification: 0.079
Achievement of MCID for HOS at 12 months	NA^a^	Baseline HOS: 0.211 BMI: 0.149 Age: 0.118 Traction time: 0.104 Alpha angle: 0.102	Labral tear: 0.218 Capsular closure: 0.113 Baseline HOS: 0.107 BMI: 0.068 Tonnis grade: 0.065 Outerbridge classification: 0.065

Abbreviations: BMI, body mass index; HOS, hip outcome score; iHOT‐12, international hip outcome tool; LASSO, Least Absolute Shrinkage and Selection Operator; LCEA, lateral centre edge angle; MCID, minimal clinically important difference; NA, not applicable.

^a^Feature importance unable to be reported for LASSO 6‐month outcomes given an AUC of 0.5.

**Table 4:** Logistic regression models.

	Achievement of the MCID for iHOT‐12 at 6 months		Achievement of the MCID for iHOT‐12 at 12 months		Achievement of the MCID for HOS at 6 months		Achievement of the MCID for HOS at 12 months	
Predictor	OR (95% CI)	*p*‐Value	OR (95% CI)	*p*‐Value	OR (95% CI)	*p*‐Value	OR (95% CI)	*p*‐Value
Age	0.993 (95% CI 0.940–1.048)	0.790	0.985 (95% CI 0.941–1.031)	0.524	1.019 (0.981–1.059)	0.331	1.017 (0.976–1.060)	0.414
Sex	1.322 (95% CI 0.530–3.294)	0.549	2.351 (95% CI 1.044–5.293)	0.039	1.602 (0.830–3.094)	0.160	1.699 (0.860–3.359)	0.127
BMI	1.001 (95% CI 0.912–1.099)	0.976	1.017 (95% CI 0.932–1.110)	0.705	0.990 (0.927–1.058)	0.771	0.975 (0.907–1.047)	0.486
Tonnis grade	0.994 (0.469–1.222)	0.255	0.672 (95% CI 0.349–1.296)	0.236	0.937 (0.600–1.564	0.775	0.999 (0.611–1.633)	0.997
Alpha angle	1.007 (95% CI 0.964–1.052)	0.754	1.009 (95% CI 0.971–1.048)	0.652	1.001 (0.970–1.032)	0.971	1.018 (0.987–1.048)	0.307
LCEA	0.979 (95% CI 0.910–1.054)	0.578	0.982 (95% CI 0.777–6.893)	0.595	1.019 (0.966–1.074)	0.491	0.997 (0.943–1.053)	0.902
Labral Tear	1.107 (95% CI 0.325–3.762)	0.871	2.314 (95% CI 0.777–6.893)	0.132	1.225 (0.407–3.686)	0.718	0.997 (0.943–1.053)	0.190
Outerbridge	0.937 (95% CI 0.595–1.475)	0.780	0.646 (95% CI 0.435–0.960)	0.030	0.863 (0.646–1.152)	0.317	1.943 (0.719–5.250)	0.064
Sporting activity	1.476 (95% CI 0.797–2.734)	0.215	1.492 (95% CI 0.812–2.744)	0.198	0.986 (0.625–1.556)	0.952	1.061 (0.661–1.704)	0.806
Traction time	1.014 (0.989–1.040	0.282	1.030 (95% CI 1.005–1.055)	0.018	1.010 (0.993–1.027)	0.236	1.018 (0.999–1.037)	0.063
Capsular closure	1.917 (95%CI 0.745–4.934)	0.178	1.106 (95% CI 0.472–2.593)	0.816	0.613 (0.311–1.207)	0.157	0.786 (0.379–1.630)	0.517
Osteochondroplasty	0.210 (95%CI 0.070–0.634)	0.006	0.628 (95% CI 0.263–1.499)	0.294	0.719 (0.378–1.370)	0.316	0.905 (0.452–1.811)	0.778
Baseline iHOT‐12	0.963 (95%CI 0.943–0.984)	0.001	0.998 (95% CI 0.976–1.020)	0.836	NA	NA	NA	NA
Baseline HOS	NA	NA	NA	NA	0.970 (0.957–0.982)	2.593E–06	0.977 (0.964–0.989)	0.0003

Abbreviations: BMI, body mass index; CI, confidence interval; HOS, hip outcome score; iHOT‐12, international hip outcome tool; LCEA, lateral centre edge angle; MCID, minimal clinically important difference; NA, not applicable; OR, odds ratio.

##### 12 months

Logistic regression achieved the best discriminatory performance, with both male sex (OR = 2.351, 95% CI: 1.044–5.293, *p* = 0.039) and increased traction time (OR = 1.030, 95% CI: 1.005–1.055, *p* = 0.018). The second highest performing model was Random forest with baseline iHOT‐12 (0.159), alpha angle (0.131), BMI (0.125), age (0.117) and traction time (0.107) as important prognostic factors (Tables 3, 4 and Figure 3).

#### HOS—MCID predictors

##### 6 months

**Figure 4:**
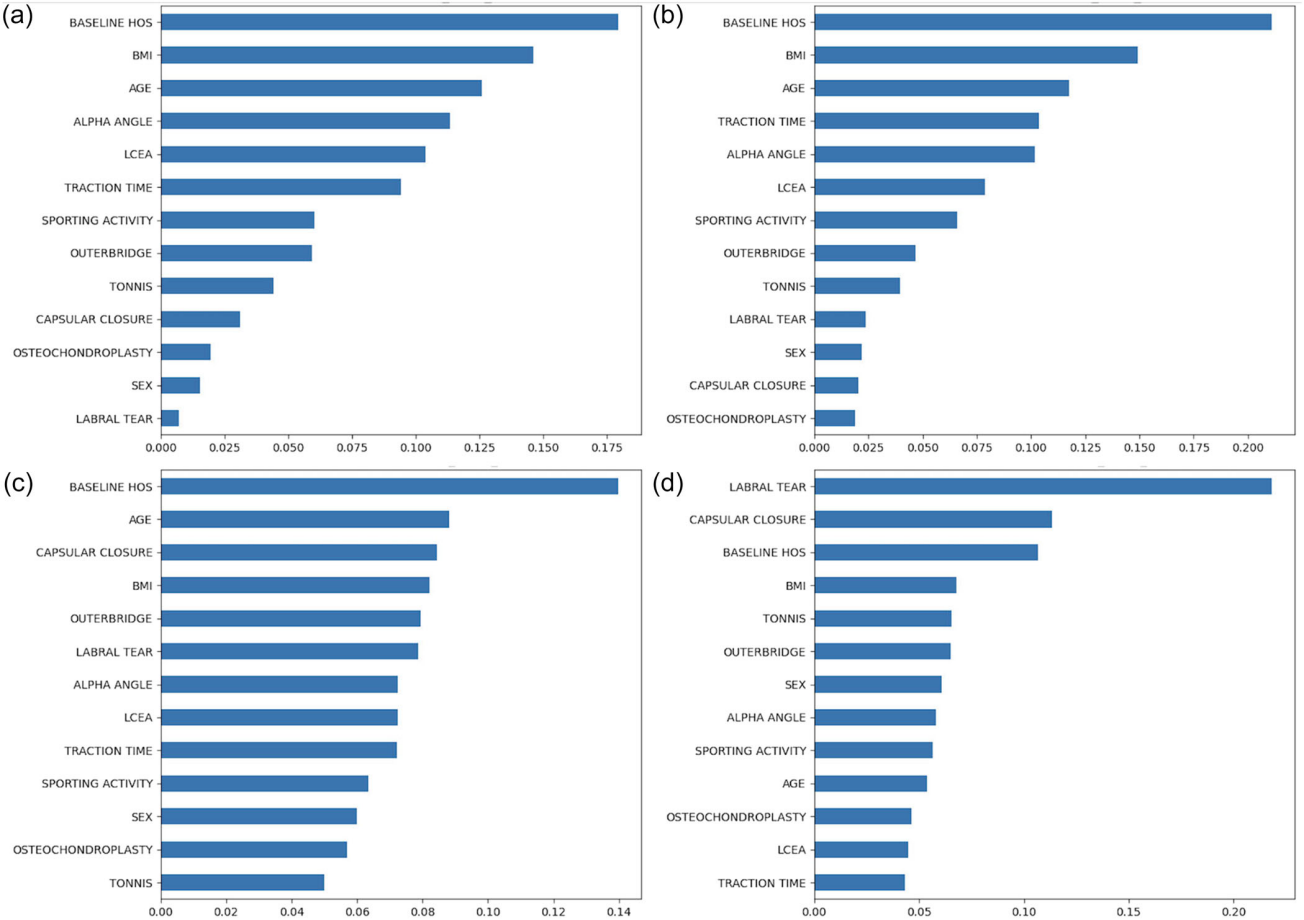
Relative feature importance for Random Forest model predicting minimal clinically important difference (MCID) of the Hip Outcome Score (HOS) at (a) 6 (top left) and (b) 12 (top right) months and XGBoost predicting MCID of the HOS at (c) 6 (bottom left) and (d) 12 (bottom right) months. Increasing scores refer to increasing importance.

LASSO demonstrated the strongest discriminatory performance, with decreased baseline HOS (–0.705), lack of capsular closure (–0.176), and male sex (0.118) being associated with MCID achievement. For logistic regression, lower baseline HOS was the only significant predictor (OR = 0.970, 95% CI: 0.957–0.982, *p* < 0.001)(Tables 3, 4 and Figure 4).

##### 12 months

XGBoost and Random Forest were the best‐performing models overall. Labral tears (0.218), capsular closure (0.113), baseline HOS (0.107), BMI (0.068), and Tonnis grade (0.065) were the best prognostic factors in the XGBoost model. Baseline HOS (0.211), BMI (0.149), age (0.118), traction time (0.104), and alpha angle (0.102) were the best prognostic factors in the Random Forest model. Logistic regression also performed well, with decreased baseline HOS (OR = 0.977, 95% CI: 0.964–0.989, *p* = 0.0003) as the only significant feature(Tables 3, 4 and Figure 4).

## DISCUSSION

The most important finding of this analysis was that lower baseline scores were associated with a greater chance of reaching MCID at 6 months and both 6 and 12 months postoperative for iHOT‐12 and HOS, respectively. All algorithms performed poorly with predicting the iHOT‐12 especially at 6 months; however, at 12 months, despite models having moderate discriminatory performance, there was poor calibration between models. For HOS, there was moderate predictive performance at both 6 and 12 months; however, calibration was better than the models for iHOT‐12. These results highlight that in certain scenarios, complex machine learning algorithms may not translate into better predictive performance than traditional logistic regression models. Furthermore, the most important prognostic variables can be used for setting realistic expectations in shared‐decision‐making discussions regarding hip arthroscopy for FAI.

Regarding feature importance, a common predictor between all models was that lower preoperative PROM scores were associated with increased chances of achieving the MCID after hip arthroscopy. Given that the definition of the MCID when using a distributive method is a fixed improvement threshold regardless of baseline, lower baseline scores have more room for improvement [20]. Those with near‐normal preoperative scores may face a ceiling effect that limits their achievable clinically significant gain [18]. A previous analysis demonstrated that lower preoperative iHOT‐12 scores were a significant predictor of achieving the MCID at 12 months postoperative [18]. Furthermore, another study reported that lower HOS scores were associated with achievement of the MCID, but did highlight that higher scores were associated with achievement of the patient acceptable symptom state (PASS), which utilizes anchor‐based methodologies [4]. A similar phenomenon is noticed in the fields of spine surgery and arthroplasty. Therefore, these findings in this study and across multiple other studies in a variety of orthopaedic fields reinforce that patients with more severe preoperatively have a greater capacity to realize a clinically important difference in PROMs [1, 8, 12, 28]. This is a consideration that orthopaedic surgeons can use when interpreting predictive models and when setting expectations with patients.

A previous study from the FIRST cohort demonstrated that increased baseline iHOT‐12 scores were associated with less absolute improvement, with an adjusted mean difference (AMD) of –0.52 (95% CI: –0.80, –0.24) per 1‐point increase in baseline scores (*p* < 0.001) [13]. Similar findings were reported in the same study for the HOS‐Sport (AMD = –0.50 [95% CI: –0.69, –0.30]) and HOS‐ADL subscales (AMD = –0.55 [95% CI: –0.72, –0.37]) [13]. This is consistent with the findings from the current machine learning and logistic regression analysis, finding that lower baseline scores were consistently associated with achieving the MCID aside from iHOT‐12 at 12 months. A previous review of 9272 hips among 39 studies found that younger age, male sex, and lower BMI, relief from diagnostic intra‐articular hip injections, and decreased osteoarthritis were all predictors of improved outcomes [27]. While lower baseline function was the most important and consistent predictor for MCID achievement, the age, sex, and BMI were all secondary predictors. Therefore, demographic and structural variables likely do contribute meaningfully to individualized outcome prediction and should be integrated into future risk stratification tools.

LASSO underperformed in three of four outcome predictions, having lower discriminatory ability compared with other models and failing to identify any statistical significant predictors of MCID achievement. The model works by shrinking less important feature coefficients or factors with nonlinear effects to zero, simplifying the model while running the risk of omitting potentially relevant predictors [32]. In other clinical prediction studies, LASSO was also outperformed by other machine learning algorithms in both discrimination and calibration. Theoretically, the advantage of tree‐based algorithms such as Random Forest and XGBoost is the capturing of complex nonlinear relationships relative to traditional linear regressions [32]. While linear regression performed either better or inferior to that of machine learning models, it is possible that this may be due to a lower sample size. For example, a previous study of 1118 patients outlined that the highest performing model using six different variables had a c‐statistic of 0.77 for predicting achievement of the HOS Sports Subscale at a minimum of 2 years postoperative [14]. Alternatively, another analysis of 1197 patients found AUCs between 0.69–0.75 for various models for the prediction of iHOT‐12 at a minimum of 6 months [23]. While discriminatory performance is important, calibration statistics are just as important in order for models to be trustworthy [29]. Poorly calibrated models may mislead clinical decision‐making, with overestimation of success causing patients to have false expectations, whereas underestimation of success causing patients to be dissuaded from surgery [29]. For example, while XGBoost and Random Forest demonstrated marginally higher AUCs than Logistic regression for HOS at 12 months, Logistic regression had near‐perfect calibration slope and intercepts, with values of 1.093 and –0.079, respectively. Therefore, while all models are not ready for clinical practice, the logistic regression model for the purposes of this study may be more reliable.

### Clinical relevance

Notably, the models predicting achievement of the MCID for iHOT‐12 overall demonstrated both poor discrimination and calibration. Alternatively, the prediction models for achieving the MCID for the HOS performed better with improved discrimination and were more reliable. One potential reason for this may be because the HOS was originally created for evaluating outcomes after arthroscopic hip surgery, whereas the iHOT‐12 was created to evaluate nonarthritic hip problems in young, active patients [2]. Furthermore, it has been shown that at the 6‐month mark, the majority of patients tend to make large improvements in iHOT‐12, whereas there is less uniform improvement in HOS [2]. This is further reinforced in this analysis where approximately 83.3% achieved the MCID for the iHOT‐12 at 6 months as opposed to 64.3% achieving the MCID for the HOS at 6 months. Furthermore, the HOS is more focused on physical function as opposed to the iHOT‐12, which also encompasses social and emotional components [2]. Previous analysis has demonstrated that the HOS correlates better with general physical function metrics than iHOT‐12 [19]. Therefore, HOS being linked closer with objective outcomes posthip arthroscopy may also be a reason for why the predictive models were superior in predicting MCID achievement than that of iHOT‐12. Clinicians can look at patients who have lower baseline PROMs as potential candidates for who may do well after hip arthroscopy.

### Limitations

This analysis has several limitations. While all data were collected prospectively, the sample size was lower relative to previous studies on this topic. This may limit model generalizability and increase the risk of overfitting. However, certain models for each time point demonstrated appropriate calibration metrics. Another limitation was that the MCID for iHOT‐12 and HOS was derived using a distribution‐based method, which is considered to be inferior to anchor‐based calculations. The time gap of approximately 6 years between the inclusion of the first patient and the last patient from the trial and cohort study also poses a limitation in that the field of hip arthroscopy continues to rapidly evolve year after year. Due to the limited sample size, predictors were selected based on clinical relevance and prior literature rather than more statistical methods such as recursive feature elimination.

## CONCLUSION

The most robust predictor of MCID achievement for both PROMs were lower baseline scores, and can be used as a prognostic variable for preoperative counselling. Model performance for predicting MCID was superior for HOS relative to iHOT‐12. Machine learning models generally had comparable discrimination and calibration scores to traditional logistic regression models.

## AUTHOR CONTRIBUTIONS

**Prushoth Vivekanantha**: Study idea conception; statistical analysis; manuscript writing and editing. **Jeffrey Kay**: Supervision; manuscript writing and editing. **Nicole Simunovic**: Supervision; manuscript writing and editing. **Olufemi R. Ayeni**: Manuscript writing and editing.

## CONFLICTS OF INTEREST STATEMENT

Olufemi Ayeni reports a relationship with Canada Research Chair in Joint Preservation that includes board membership. Olufemi Ayeni reports a relationship with Journal of ISAKOS that includes board membership. Nicole Simunovic reports a relationship with Journal of ISAKOS that includes board membership. The remaining authors declare no conflicts of interest.

## ETHICS STATEMENT

The authors have nothing to report.

## Supporting information

SUPPLEMENTARY DIGITAL MATERIAL.

## Data Availability

Data may be made available upon reasonable request at prushoth.vivekanantha@medportal.ca.
