# Supplementary material for: Lower baseline scores best predict achievement of the minimal clinically important difference after hip arthroscopy: A machine learning analysis from the Femoroacetabular Impingement RandomiSed Controlled Trial and embedded prospective cohort
Source: Knee Surg Sports Traumatol Arthrosc. 2025 Sep 9;33(12):4385–97. doi: 10.1002/ksa.70053 (PMC12684352; doi:10.1002/ksa.70053)
Supplement: Supplementary file 1 — SUPPLEMENTARY DIGITAL MATERIAL. [file KSA-33-4385-s001.docx]

**SUPPLEMENTARY DIGITAL MATERIAL**

**Table 1.** Artificial intelligence and model evaluation terms

| **Terminology** | **Definition** |
| --- | --- |
| Machine Learning | AI-based algorithms that learn patterns from data to make predictions |
| Area Under the Curve | Evaluates discrimination, the ability for a model to distinguish between true-positive and false-positive cases - values of 0.5 indicates the model performing no better than chance, values of 1.0 indicates perfect discrimination |
| Calibration | Evaluates model risk estimates and how well in accordance they are with true observed values  *Calibration slope:* Represents if predicted values are too moderate or too extreme (1 = perfect calibration)  *Calibration intercept:* Represents if the model on average overestimates or underestimates prevalence (0 = perfect calibration) |
| Brier Score | An extension of calibration analysis, evaluates the mean squared difference between true outcomes and corresponding predicted probability risks - values of 0 indicate total accuracy, values of 1 = complete inaccuracy |

AI = artificial intelligence, all definitions from Polce et al.[[26]](https://www.zotero.org/google-docs/?iz0W1A)

**Figure 1.**


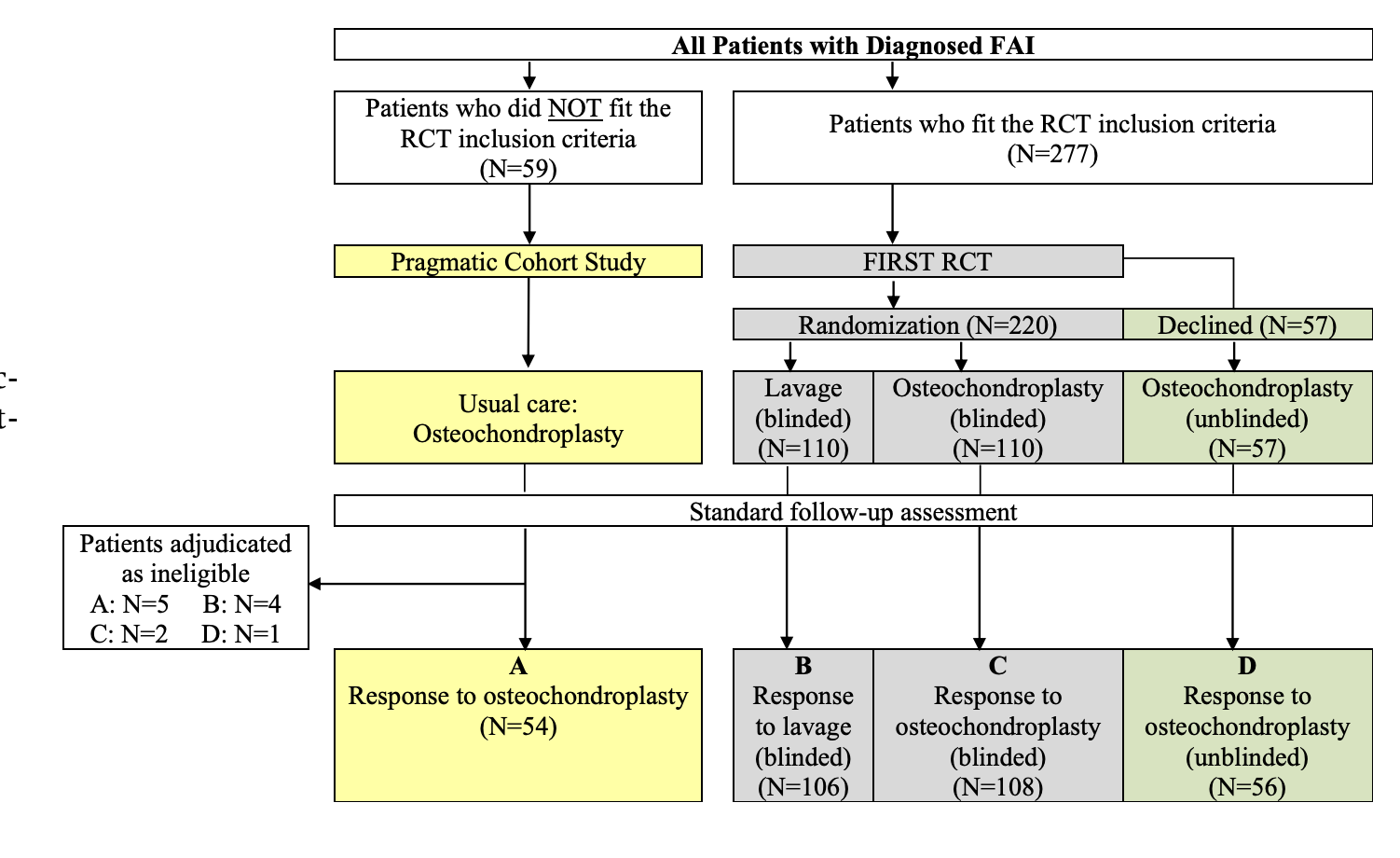


Flowchart for initial FIRST trial and Embedded Prospective Cohort [[1]](https://www.zotero.org/google-docs/?fWpFPG)
